# Short LOV Proteins in *Methylocystis* Reveal Insight into LOV Domain Photocycle Mechanisms

**DOI:** 10.1371/journal.pone.0124874

**Published:** 2015-05-01

**Authors:** Kaley K. El-Arab, Ashutosh Pudasaini, Brian D. Zoltowski

**Affiliations:** Department of Chemistry, Center for Drug Discovery, Design and Delivery, Southern Methodist University, Dallas, Texas, United States of America; University of Texas at Austin, UNITED STATES

## Abstract

Light Oxygen Voltage (LOV) proteins are widely used in optogenetic devices, however universal signal transduction pathways and photocycle mechanisms remain elusive. In particular, short-LOV (sLOV) proteins have been discovered in bacteria and fungi, containing only the photoresponsive LOV element without any obvious signal transduction domains. These sLOV proteins may be ideal models for LOV domain function due to their ease of study as full-length proteins. Unfortunately, characterization of such proteins remains limited to select systems. Herein, we identify a family of bacterial sLOV proteins present in *Methylocystis*. Sequence analysis of Methylocystis LOV proteins (McLOV) demonstrates conservation with sLOV proteins from fungal systems that employ competitive dimerization as a signaling mechanism. Cloning and characterization of McLOV proteins confirms functional dimer formation and reveal unexpected photocycle mechanisms. Specifically, some McLOV photocycles are insensitive to external bases such as imidazole, in contrast to previously characterized LOV proteins. Mutational analysis identifies a key residue that imparts insensitivity to imidazole in two McLOV homologs and affects adduct decay by two orders of magnitude. The resultant data identifies a new family of LOV proteins that indicate a universal photocycle mechanism may not be present in LOV proteins.

## Introduction

Light Oxygen Voltage (LOV) domain containing proteins enable organisms to adapt to alterations in environmental stimuli including blue-light, oxygen sensing, and stress responses. LOV proteins are typically modular in nature, where the stimuli-responsive LOV domain is coupled to N- or C-terminal signal transduction elements [1–3]. The photoactive LOV domain then couples changes in environmental variables to affect small molecule chemistry. In LOV domains this specifically involves the absorption of blue-light by a flavin (FMN or FAD) cofactor to form a covalent adduct with a conserved Cysteine residue. Blue-light induced formation of the flavin C4a adduct then enables allosteric regulation of signal transduction elements to regulate diverse biological function [4]. For instance, LOV proteins are involved in blue-light regulation of gene expression in *Neurospora crassa* [5,6] as well as entrainment of circadian clocks, phototropism, and flowering in *Arabidopsis thaliana [7]*. In bacteria, LOV proteins have been shown to regulate pathogenicity and adaptation to environmental stress [8–11]. The wide range of functional outputs regulated by LOV domain chemistry has led to the advent of their usage in the field of optogenetics. In these optogenetic systems, the LOV domain imparts photodynamic control of biological function through the design of engineered proteins [12]. Currently the fidelity of LOV-domain based photoreceptors is limited in part by incomplete understanding of chemical and structural components of LOV signaling.

Typically LOV proteins have been characterized as being modular in nature with photoresponsive LOV components coupled to a wide array of signal transduction elements, including phosphodiesterases, transcription factors, F-box proteins and histidine kinases [2,4,5,11,13–15]. More recently, short LOV (sLOV) domains have been identified [16–19]; these harbor only the photoactive LOV domain, thereby lacking the N- or C-terminal effector domains important for signal transduction. These are most typically found in bacteria, where sLOV proteins compose 13% of all LOV proteins [18]. Despite their widespread nature in bacteria, the mechanism of action for sLOV proteins remains poorly explored and is limited to select systems.

Currently, the best model of sLOV signaling exists in fungal systems, where homologs of the sLOV protein VVD are involved in circadian function and adaptation to increasing levels of blue light [20]. In filamentous fungi, circadian regulation of gene transcription is achieved via competitive dimerization of two LOV containing proteins, VVD and White Collar-1 (WC1) [5,13,21]. In this system, the sLOV protein VVD bears homology to a LOV domain in WC1. Photoactivation of both proteins leads to both homo- and hetero-dimerization [5,22–24]. WC1 homodimers are active for gene transcription; however, heterodimerization with VVD dampens transcriptional activity [5,6]. In this manner, the sLOV protein can affect gene transcription through a process of competitive dimerization. Essential to such a model is strong homology between the sLOV protein and a LOV domain in a signal-transduction protein that requires dimerization for function.

Recently, we have identified an analogous regulatory network involving the sLOV protein ENVOY (ENV1) in *Trichoderma reesei*. In *T*. *reesei*, ENV1 acts in an equivalent signaling node to VVD, although with distinctly altered mechanisms [25–28]. In ENV1, a two-residue shift in the position of a key Cysteine residue enables integration of both light and oxidative stress [25]. Sequence conservation of ENV1 and VVD in fungal circadian clocks suggests a competitive dimer model of sLOV signal transduction may be more widely conserved in biology, where sLOV proteins may enable both light responsive and oxygen sensitive signaling.

**Fig 1:**
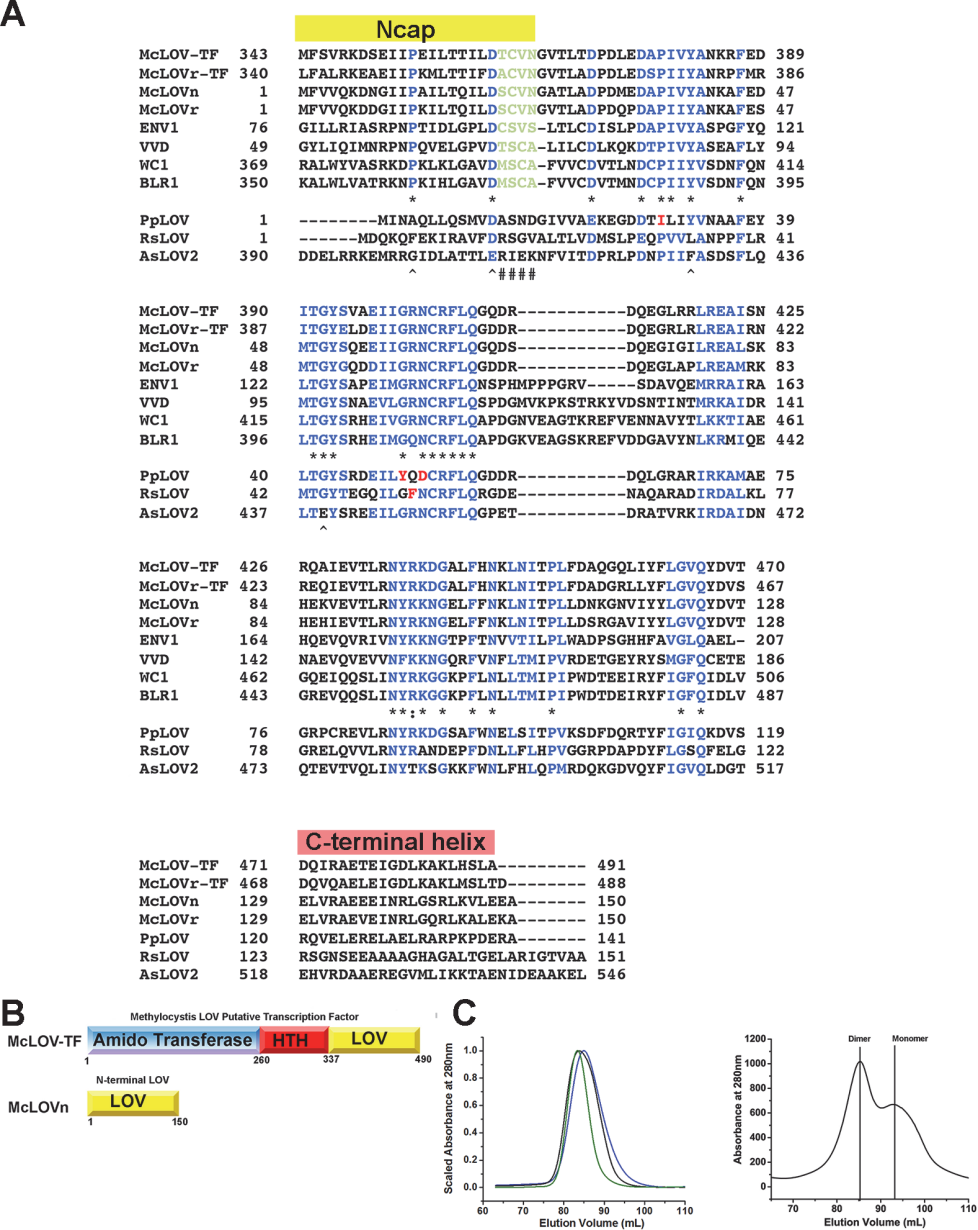
Model for competitive dimerization. (A) Sequence alignments of LOV proteins in *Methylocystis* compared to fungal analogues and the LOV 2 domain of *Avena sativa* Phototropin (AsLOV2). Comparisons of a LOV-transcription factor in *Methylocystis* (McLOV-TF) and *Methylocystis rosea* (McLOVr-TF) demonstrate high homology to sLOV proteins in the respective organisms (McLOVn, McLOVr). Key elements required for signal transduction are conserved in the fungal sLOV proteins *Trichoderma reesei* ENVOY (ENV1) and *Neurospora crassa* Vivid (VVD) as well as fungal LOV transcription factors *N*. *crassa* White-Collar 1 (WC1) and *T*. *reesei* Blue-light receptor 1 (BLR1). These elements are not conserved in AsLOV2 or short LOV domains from *P*. *putida* (PpLOV) or *R*. *sphaeroides* (RsLOV). Specifically, a key hinge region is conserved in all bacterial and fugal proteins (light green) within the NCap. Two additional residues are absolutely conserved that form key contacts in organization of the NCap hinge region. Core signaling regions of the LOV domain (blue) are conserved in all species. Residues absolutely conserved in all species (*) are noted, residues conserved in fungal and bacterial species, but not AsLOV2 are shown (^) as well as strongly conserved elements (:). (B) Domain architecture in *Methylocystis* LOV proteins. (C) SEC of McLOVr (black), McLOVn (green) and 337-TF (blue). All elute as dimers with apparent molecular weights (MW) of 35 kDa (McLOVr), 33.5 kDa (McLOVn) and 32 kDa (337-TF). The expected MW of a monomer was 17 kDa. Some preparations of McLOVn contain a significant monomeric fraction. Two distinct peaks with apparent MWs of 34 kDa and 17 kDa are observed.

Herein, we identify an analogous system of LOV proteins in the bacteria *Methylocystis*. Genome analysis of *Methylocystis* species identifies conservation of two LOV domain-containing proteins (Fig 1). One is a sLOV protein that bears homology to *T*. *reesei* ENVOY (ENV1). The second is a modular LOV protein housing an N-terminal ARAC domain coupled to both a Helix-turn-Helix (HTH) and C-terminal LOV domain. A high degree of homology between the sLOV protein and the C-terminal LOV component suggests mechanisms analogous to fungal signaling networks. Cloning, expression, and characterization of *Methylocystis* LOV proteins confirms functional dimer formation and unique chemical signaling mechanisms. Specifically, kinetic and thermodynamic characterization of *Methylocystis* LOV photocycles reveal a residue substitution that can block base catalysis, thereby indicating that functional studies of diverse sLOV proteins may shed light on alternative photocycle pathways and signaling mechanisms that may aid in the improved design of optogenetic devices.

## Results

In the fungal sLOV model, the key features facilitating competitive dimerization are a LOV transcription factor (LOV-PAS-Zn-finger; LOV-TF) and a sLOV protein that exhibit homology within their respective LOV domains. We recently identified a similar signal transduction pathway in *Trichoderma* that links stress responses, metabolism, and blue-light signaling [25,29]. To search for similar signal transduction pathways in bacterial species we searched for sLOV proteins with high homology to ENVOY (ENV1) from *Trichoderma reesei*.

Genome analysis revealed that *Methylocystis* bacteria contain two LOV proteins with moderate homology to ENV1, including the presence of a key Cysteine residue required for signal transduction in fungal sLOV proteins (Fig 1A and 1B) [25]. These include a LOV-TF containing an Amido-transferase (ARAC), Helix-Turn-Helix (HTH) and LOV domains (McLOVn-TF), and a sLOV protein (McLOVn) that retain high homology within their respective LOV domains (40% Identity, 56% similar). These fit our candidate search as LOV-HTH proteins typically require dimerization to regulate gene transcription [30]. Closer examination of sequence alignments of *Methylocystis* LOV proteins in relation to fungal competitive dimerization pathways, as well as the LOV2 domain of *Avena sativa* Phototropin 1 (AsLOV2) and other sLOV proteins in *Pseudomomas* and *Rhodobacter sphaeroides* reveals intriguing regions of conservation. In particular, fungal LOV proteins signal through conformational changes within a motif N-terminal to the core LOV domain (NCap) [3,23–25]. In the Ncap, a short turn motif involving a Cys residue required for signal transduction is conserved in fungal LOV proteins and those in *Methylocystis* (Fig 1A). In contrast, the turn motif (similar residues encompassing residues 21–24 (McLOVr numbering), including a Cys residue), as well as two additional NCap residues (Pro12, Asp20) are absolutely conserved in *Methylocystis* and fungal LOV proteins. The turn motif and Pro12 are not present in AsLOV2 or other sLOV proteins. Although McLOV proteins contain a C-terminal Jα helix that may be involved in signaling, the helix bears poor homology to those in sLOV proteins and AsLOV2. In these regards, *Methylocystis* LOV proteins retain elements required for regulation of transcription through competitive dimerization in fungal LOV proteins. Based on these similarities we became interested in examining the solution characteristics and photochemistry of sLOV proteins in *Methylocystis*.

To examine mechanisms of signal transduction within *Methylocystis* LOV proteins, we cloned, expressed, and characterized McLOVn and McLOVn-TF, as well as a McLOVn homolog from the related bacterium *Methylocystis rosea* (McLOVr*)* (Fig 1). We focused primarily on two goals. 1) Certify that the LOV domains are competent for dimerization. 2) Verify the proteins are functional LOV domain containing photoreceptors and provide detailed photochemical characterization.

Initial cloning of LOV proteins in *Methylocystis* focused on the isolated LOV domains from McLOVn and McLOVn-TF and the corresponding sLOV protein in *M*. *rosea* (McLOVr). McLOV proteins were cloned from genomic DNA and expressed in *E*. *coli*. Low expression of the proteins from genomic DNA led us to examine related proteins in *M*. *rosea* and design codon optimized constructs for *E*. *coli* expression. Due to improved protein expression from these constructs, most protein was obtained using codon optimized DNA. Further, although ideally we would like to study the transcription factor as a full-length (FL) construct, the FL protein expressed as insoluble inclusion bodies and was rather studied as the isolated LOV domain. Thus, McLOVn-TF was N-terminally truncated leaving a construct composed of residues 337–490 (337-TF) (Fig 1B). These proteins were studied in detail to reveal the oligomeric state, adduct lifetimes, and photocycle properties. The combined data suggests that currently presumed photochemical mechanisms may not be retained in all LOV proteins particularly with respect to the involvement of a base-catalyzed reversion process.

### McLOV proteins are dimeric

**Fig 2:**
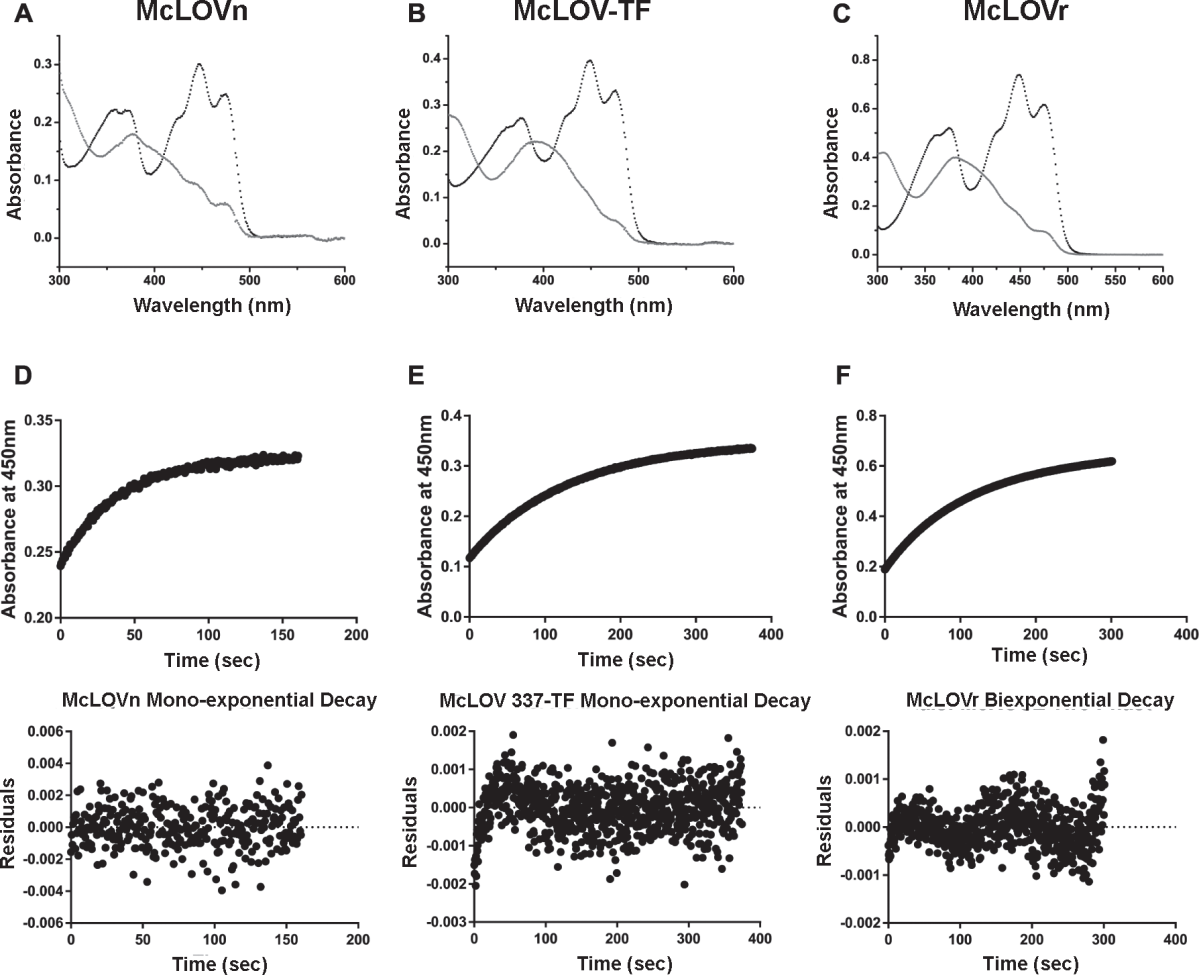
McLOV photocycles and kinetics. (A-C) McLOV proteins all demonstrate spectra consistent with C4a adduct formation. Dark-state spectra (black) are consistent with oxidized flavin. Illumination with blue light bleaches the 450 nm absorption bands (red). Subtle differences in degrees of photoactivation in McLOVn (A), 337-TF (B) and McLOVr (C) are indicated by residual presence of absorption bands centered around 450 with vibrational bands at 425 and 478 nm. (D-F) McLOVn (D), 337-TF (E) demonstrate first order kinetics as demonstrated by an absorbance trace at 450 nm and their respective residual plots. In contrast McLOVr (F) is best fit with a biexponential function.

All constructs readily purify from *E*. *coli* with a bound flavin cofactor and demonstrate spectra consistent with formation of a C4a adduct (Fig 2A–2C). Size exclusion chromatography (SEC) of LOV constructs reveals that McLOVn, McLOVr, and 337-TF are constitutively dimeric under dark state conditions (Fig 1C). Interestingly, in some McLOV purifications, a monomeric peak is present even at very high concentrations (Fig 1C). Such behavior has been observed in fungal LOV proteins that demonstrate sensitivity to oxidative stress [23]. We have been unable to determine the cause of a monomeric fraction in some preparations so far; however, conservation of key Cys residues that impart sensitivity to oxidative stress in other systems may be involved [23,25]. Thus, all *Methylocystis* LOV proteins are competent for dimerization and may partake in a model of competitive dimerization. Unfortunately, the photocycle lifetimes of all constructs were too fast to resolve any differences in oligomeric status following photo-excitation (see below). The limitations of the fast photocycles may be remedied through the design of rate altering variants, however that first requires a more thorough understanding of the LOV photocycle. Such knowledge may lead to the design of rate-altering variants for future structural studies.

### Photochemistry of LOV proteins from *Methylocystis*

All constructs purify with bound flavin and exhibit photobleaching in response to blue light (Fig 2). The dark-state spectra are consistent with an oxidized flavin with a broad absorption at 450 nm and shoulder peaks at 425 and 478 nm. The peak at 450 nm is the result of a S_0_-S_1_ electronic transition typical of the oxidized flavin. There is an additional absorption band in the UV-A region around 350 nm due to a S_0_-S_2_ electronic transition that facilitates adduct scission under illumination with broad spectrum light sources. Light excitation bleaches the 450 nm absorption band leading to a single peak centered around 390 nm, consistent with blue-light induced formation of a cysteinyl-flavin C4a adduct. In the *Methylocystis* LOV constructs, we are unable to completely form the light-state adduct; rather, illumination generates an equilibrium mixture between light- and dark-adapted states [7]. The different degrees of photoactivation likely result from different quantum yields of adduct formation as well as the rapid recovery observed in these proteins. To further clarify factors affecting LOV photocycles in *Methylocystis* species, we conducted a comprehensive analysis of the kinetics of adduct decay in all constructs.

### *Methylocystis* LOV proteins are fast-cycling

Studies of LOV proteins have revealed that the rate of adduct decay under dark-state conditions can range from seconds to days [31]. In turn the variance in adduct lifetimes can affect sensitivity to light fluence that dictates the steady-state ratio between light and dark-adapted states [7]. Kinetic analysis of LOV proteins in *Methylocystis* reveals adduct decay proceeds with first order kinetics in all constructs tested (Fig 2). Rate constants and time constants for all constructs can be found in Table 1. The rate constants of these species are quite fast compared to fungal species, but consistent with those observed in fast cycling phototropins [2,31]. Specifically, the isolated sLOV protein McLOVn exhibited mono-exponential decay with a light-state lifetime of 38 seconds. McLOVr typically displays biexponential kinetics with a fast component (80 seconds) comprising 43% of the species and a slow component (180 seconds) 57% of the species. For Arrhenius and Eyring analysis we used the fast component (see below). The adduct lifetime of 337-TF was 130 seconds (Table 1), similar to the average time constant for McLOVr. Given the high homology to slow cycling fungal LOV proteins, we set out to examine what factors led to the fast photocycle. We specifically focus on two experiments. 1) Eyring and Arrhenius parameters for adduct decay. 2) The effect of small molecule bases on adduct recovery. Previous research in other LOV proteins indicates that solvent access to the active site can be probed using small molecule bases.

**Table 1:** Kinetics of thermal reversion for LOV constructs and variants at 296 K.

Construct	Time Constant (s)	Δ^‡^	ΔS^‡^	Protection factor (a)
McLOVn	38 +/- 3	51 kJ/mole	-102 J/mol*K	NA
McLOVr	80 +/- 5 (fast 43%) 180 +/-5 (slow)	53 kJ/mole	-104 J/mol*K	NA
337-TF	130 +/- 5	71 kJ/mole	-44 J/mol*K	NA
McLOVr T27I	12300 +/- 100	-	-	30

### McLOV photocycle kinetics are entropically compensated

**Fig 3:**
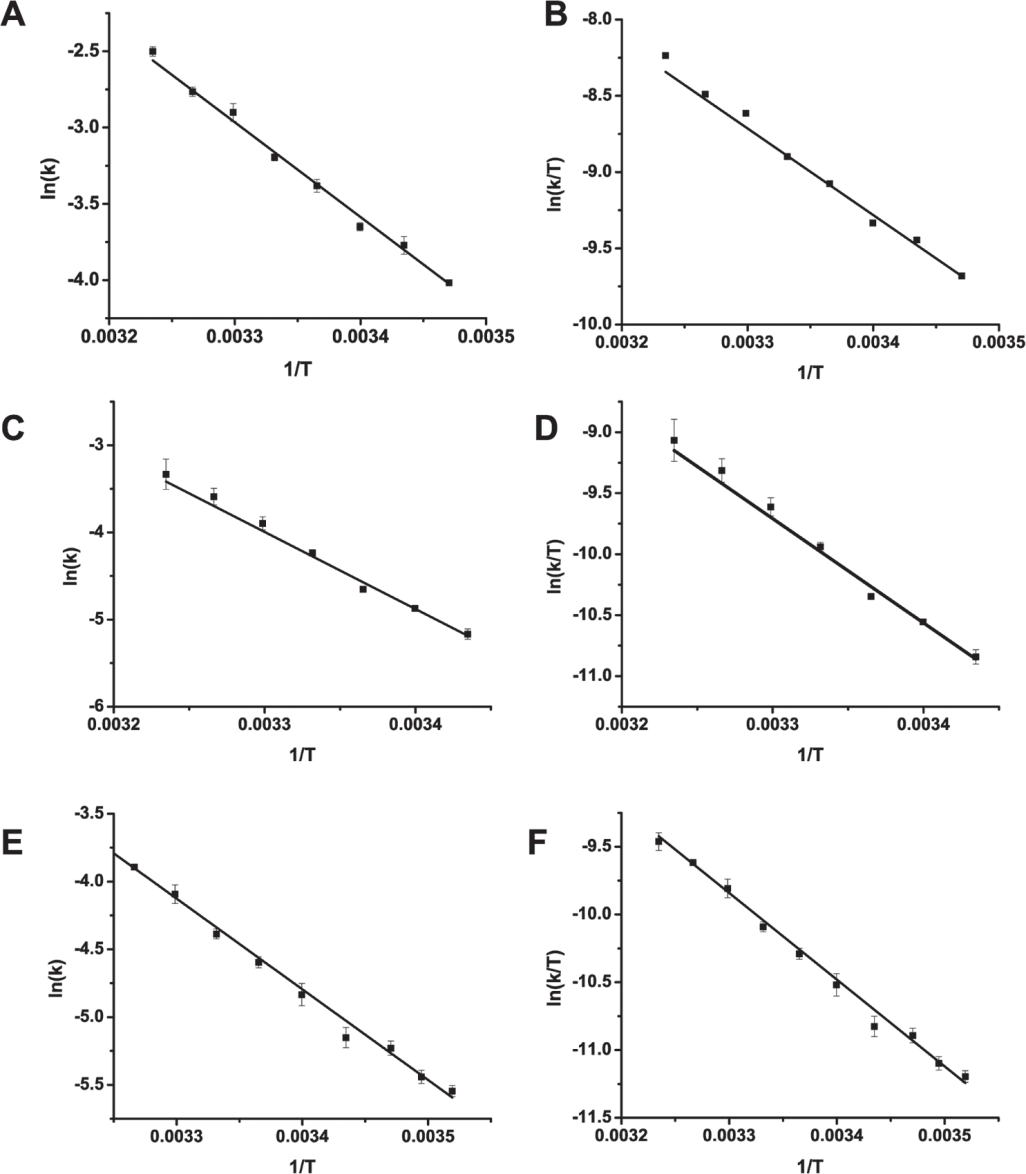
Arrhenius and Eyring analysis of McLOV proteins. Arrhenius (A,C,E) and Eyring (B,D,F) of McLOVn (A,B), 337-TF (C,D), and McLOVr (E,F) constructs. All measurements were carried out in triplicate, and error bars are shown as standard deviations relative to the mean. McLOVn and McLOVr both depict weak temperature dependence that indicates low entropies of activation with entropic compensation. In contrast 337-TF, has markedly increased enthalpies of activation (71 kJ/mole vs. ~50 KJ/mole) with a decrease in the entropic penalty (-44 J/mole*K vs. ~ -100 J/mole*K).

To better understand the photocycles of these sLOV proteins, we performed Arrhenius and Eyring analysis of dark state recovery. Typical energies of activation for fast cycling LOV domains (t1/2~30–1000 sec) are on the order of 70–100 kJ/mol [7]. In contrast, the temperature dependence of McLOVn kinetics reveals an uncharacteristically low activation energy of 52 kJ/mol (Fig 3). Eyring analysis indicates the low enthalpy of activation (51 kJ/mol) is compensated by a large unfavorable entropy of activation (-102 J/mol*K), allowing for a low energy of activation and weak temperature dependence of the rate of adduct scission (Table 1 and Fig 3). Such entropic compensation has been observed in circadian clock photoreceptors, where it may function to decrease the temperature dependence of photocycle kinetics [7]. Similarly, the McLOVn homolog McLOVr exhibits a low enthalpy of activation (53 kJ/mol) that is compensated by an unfavorable entropy of activation (-104 J/mol*K). Such similarities in the energetic parameters of adduct scission suggest evolutionary conservation of residues dictating the energy landscape during adduct scission. Given the high degree of similarity between McLOVn and the 337-TF (58% identical, 77% similar), we expected 337-TF to also exhibit a low enthalpy of activation that is entropically compensated; however, 337-TF demonstrates a markedly higher enthalpy of activation (71 kJ/mole) with a modest unfavorable entropy of activation (-44 J/mol*K). To examine whether these unique energetic parameters reflect additional alteration to the reaction landscape, we examined all constructs for the effect of small molecule bases on adduct decay.

### External bases do not catalyze McLOVn and McLOVr

Scission of the Cysteinyl-C4a adduct has been shown to be catalyzed by small molecule bases such as imidazole [32]. These base catalysis studies in combination with solvent-isotope effects have led to a general model of adduct decay, where abstraction of the flavin N5 proton is rate limiting [31,32]. Currently, the intrinsic base is unknown. Notably, although LOV proteins are catalyzed by small molecule bases, the dependence of adduct decay on base concentration varies widely within the LOV family. Differences in sensitivity to base catalysis can often be explained via differences in solvent access to the active site, where an increase in solvent accessibility correlates to faster photocycles kinetics and sensitivity to small molecule bases [31,33]. We wanted to examine similar phenomena in McLOV proteins.

**Fig 4:**
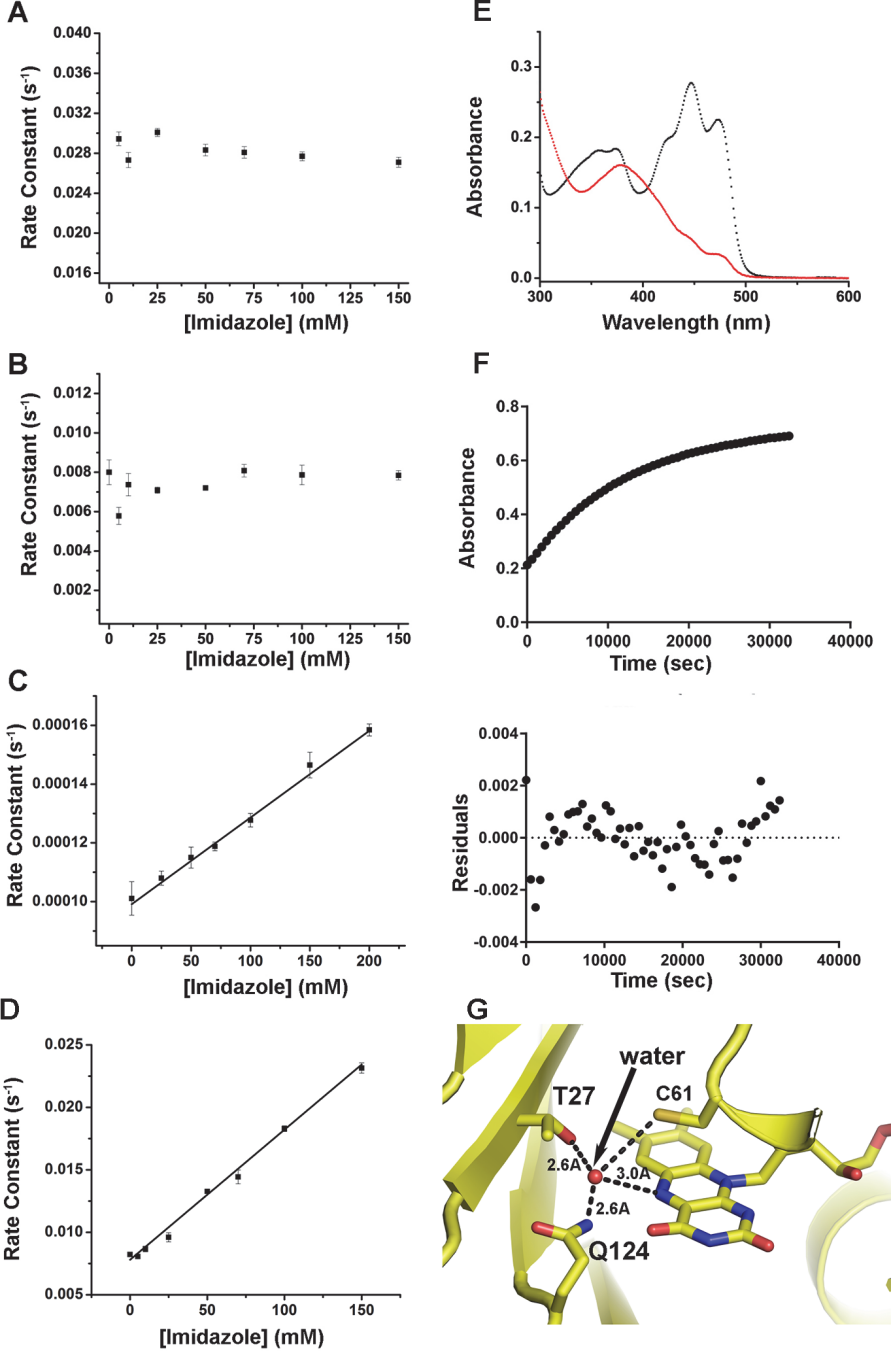
External bases do not catalyze some McLOV proteins. (A,B) Both McLOVn (A) and McLOVr (B) show no dependence of adduct decay on imidazole concentration. In contrast, a Thr27Ile demonstrates spectra consistent with LOV chemistry (E) with a 100-fold decrease in the rate of adduct decay (F). The Thr27Ile variant causes an imidazole concentration dependent rate of adduct scission (C). Thus, Thr27 renders McLOV proteins insensitive to imidazole catalysis. (D) In contrast, 337-TF is imidazole sensitive despite containing a Thr at a position equivalent to 27. (G) A LOV structure (modeled from ENVOY PDB: 4WUJ) containing a Thr at a position equivalent to 27. Thr27 can coordinate a hypothetical water molecule between the N5 position on flavin, C61 and Q124 (McLOVr numbering). In all figures, error bars represent the standard deviation of the mean for experiments conducted in triplicate.

In contrast to other LOV domains, the dark state reversion process is not base catalyzed in McLOVn or McLOVr (Fig 4A and 4B). Addition of imidazole concentrations up to 150 mM has no effect on the rate of adduct scission at pH 8.0. Such behavior is surprising as most LOV proteins demonstrate remarkable base catalysis at 10–100 fold lower concentrations [7,34]. Even more surprising, despite high sequence conservation 337-TF demonstrates a large dependence of adduct decay on external bases (Fig 4).

Several factors could contribute to the divergent dependence of adduct decay on small molecule bases. These include: 1) No involvement of proton abstraction in the rate determining step. 2) A lack of solvent accessibility to the protein active site. We exclude this as a possibility, due to the fact that exclusion of solvent from the active site would lead to a slow rate of adduct decay, which is not observed. 3) The presence of an intrinsic base that renders external bases ineffective at accelerating adduct decay. To examine these factors further we conducted solvent isotope studies, as well as examined McLOV proteins for residues that could attenuate solvent accessibility or function as an intrinsic base.

Solvent isotope studies have been widely used in LOV proteins to examine the kinetics of adduct formation and adduct scission. SIEs have been reported of up to 5 for adduct formation and 2–4 for adduct decay [31,33,35]. Proton inventory studies confirm that a single proton transfer event, presumably involving N5 of the isoalloxazine ring, is rate limiting [31]. If such a proton transfer event is not rate limiting in McLOVn, McLOVr, and 337-TF, then we should observe little or no SIE. In contrast, McLOVn and McLOVr demonstrate SIEs of 3.4 and 2.5, respectively. Both values are consistent with a primary SIE, indicating that N5 deprotonation likely remains rate-limiting in these systems.

Since SIEs are consistent with N5 deprotonation being rate limiting, and the adduct decay rate is inconsistent with solvent exclusion from the active site, we presume that an intrinsic base may attenuate base catalysis in these systems. To probe such a possibility we examined McLOVn, McLOVr, and 337-TF sequences for residues that could either function as an intrinsic base or facilitate proton transfer from N5. A candidate residue was identified adjacent to the active site flavin and a conserved Gln residue required for LOV function. McLOVn, McLOVr, and 337-TF contain a Thr residue at position 27 (McLOVr numbering), where LOV proteins typically contain a hydrophobic residue (lle or Val) [31]. The site is well positioned adjacent to the N5 position, creating a small pocket lining N5 of the isoalloxazine ring, conserved Gln124 (McLOVr numbering), and Thr27 (Fig 4). Consistent with a role in affecting adduct decay, a Thr27Ile variant leads to a 100-fold decrease in the rate of adduct decay (Table 1 and Fig 4). In addition, a Thr27Ile variant modestly restores base catalysis in adduct decay (Fig 4C). Specifically, an increase of imidazole from 0 mM to 200 mM leads to a greater than 2-fold effect on the rate of adduct decay, consistent with a solvent-accessibility factor of 30. Such a value is consistent with weak base-catalysis as observed in other circadian clock LOV domains (ZTL, VVD) [7]. Notably, the high imidazole sensitivity of 337-TF indicates that a Thr at position 27 is not the sole factor influencing base catalysis, as 337-TF demonstrates a large solvent accessibility factor (a = 10000). We conclude that a unique residue substitution in the vicinity of the flavin N5 position greatly alters base catalysis, including rendering some sLOV proteins insensitive to base catalysis. Thus, examination of sLOV proteins in disparate systems indicates that mechanisms of LOV photocycles may not be universally conserved.

## Discussion

LOV domain chemistry has been studied in detail in several systems [31–33,36]. Although some debate in regards to photocycle intermediates remains, a consensus mechanism involves the formation of a radical intermediate [2,35,37–39]. In LOV proteins, excitation with blue-light allows formation of a flavin singlet state that rapidly undergoes intersystem crossing. The resultant triplet state then induces electron transfer, presumably from the active site Cysteine, to form a semiquinone intermediate. Proton transfer from the Cys residue to the N5 position of the isoalloxazine ring is coupled to radical recombination resulting in formation of the C4a-adduct signaling state. Upon return to the dark, the adduct spontaneously decays on the order of seconds to days to allow sensitivity to environmental light fluence [7]. Currently, research has examined the adduct-lifetime and discovered several factors key to stabilization of the signaling state. These include: 1) Alteration of the flavin reduction potential to favor reduced semiquinones [31]. 2) Steric stabilization of the C4a adduct [40]. 3) A decrease in solvent accessibility to the N5 position of the isoalloxazine ring [31] and 4) Alteration of H-bonding networks to the active site flavin [7,33,36]. The latter two are believed to affect the rate limiting abstraction of the N5 proton by endogenous or exogenous bases, however the precise mechanism of proton abstraction remains elusive.

Herein we have identified a family of LOV proteins in bacteria that may provide insight into signal transduction by sLOV proteins as well as variability in the photocycle landscape across the LOV family. sLOV proteins within *Methylocystis* bear homology to LOV-TF proteins, both of which exist as constitutive dimers under dark-state conditions. These proteins may in turn be capable of dictating light responses through regulation by competitive dimerization as observed in fungal systems. Moreover, chemical characterization of these proteins provides evidence to the natural base-catalysis mechanisms that may dictate LOV photocycle kinetics.

### Implications for base catalysis

To the best of our knowledge, all LOV proteins that have been characterized demonstrate adduct decay pathways that are base catalyzed. In contrast, McLOVn and McLOVr are insensitive to external base concentrations. Examination of the LOV active site revealed an active site Thr that is well positioned to either activate a conserved glutamine as an intrinsic base, or to recruit and coordinate a base to the protein active site (Fig 4G). In McLOVn and McLOVr, the presence of Thr27 is essential to the abolishment of base catalysis and contributes dramatically to the fast photocycle present in these proteins. In contrast 337-TF exhibits strong base catalysis even in the presence of a Thr at the equivalent position. We propose that the Thr27 position directs the role of exogenous (imidazole) and endogenous bases in LOV catalysis. The divergent behavior in McLOV proteins in turn is likely coupled to nearby residues or alteration of N- C-terminal extensions to the LOV core. Such large effects on imidazole catalysis have been observed in other systems [34]. We further propose that the divergent dependence on imidazole allows McLOV proteins to function as model proteins for understanding the base-catalyzed mechanism present in all LOV proteins. We specifically address two proposed theories involving base catalysis in light of our data on McLOV proteins.

Small bases such as imidazole have been previously proposed to facilitate proton abstraction from the light state adduct [32]. These studies identified two plausible mechanisms for proton abstraction. The first suggests that an ordered solvent channel conserved in all LOV structures functions as a proton shuttle [41]. In such a model, imidazole acts as an external base that activates peripheral ordered water to facilitate proton transfer from N5. The second suggests that imidazole can act directly from the active site to abstract the N5 proton [31]. The second mechanism is supported by proton inventory measurements that implicate a single proton transfer event in the rate determining step in adduct decay. The mechanism is further supported by residue substitutions in the fungal LOV protein VVD that removes steric hindrance present in the LOV active site [31]. These substitutions enhance base catalysis in VVD and lead to a concentration dependence that fits a single site saturation model. Importantly, other LOV proteins demonstrate unsaturateable base catalysis. Even VVD variants that exhibit saturation must be modeled with a second unsaturateable component. In these regards, a direct site of action may not be a complete model.

Alternatively, the first mechanism proposes that ordered water directs base catalysis. Although known LOV structures conserve an ordered solvent channel leading to the flavin active site, no ordered water has been observed within H-bonding distances to N5 [41]. Further, such a mechanism contradicts proton inventory studies in several LOV systems. Thus, such a mechanism may not be accurate.

Based on our studies here, we propose an alternative hybrid mechanism. We propose that ordered water may function as an intrinsic base in LOV proteins. However, the ordered water near N5 is transient in nature and cannot be observed in existing LOV structures. The transient ordered water could then be activated by imidazole that acts in a single site of low affinity near the periphery of the conserved solvent channel. Such a mechanism is consistent with existing mutational data in VVD that requires mutation of an Ile residue near the periphery of the solvent channel to impose a saturateable model [31]. Moreover, introduction of an active site Thr in McLOV proteins may increase the occupancy of ordered water near N5 rendering base catalysis pathways comparatively inefficient. In such a system, water ordered by Thr27, Gln124, and N5 are directly activated by the surrounding protein, thereby out competing a very low affinity site for base catalysis. Importantly, such a function would also be dependent on the orientation of Gln124 as well as interactions to the N/Ccaps. Thus, Thr27 acts in a key position to have the capacity to alter imidazole catalysis in divergent manners. Importantly, a lack of crystal structures of McLOV proteins renders examination of ordered water in LOV proteins complicated. Future structural studies may shed light on the role of ordered water in base catalysis of LOV proteins. Combined, our studies highlight the importance of studying LOV proteins from novel environmental niches as they can facilitate our understanding of the global LOV photocycle mechanism as well as allosteric regulation of LOV function. Such studies can shed light on new mechanisms for optogenetic tool development and improvement of existing optogenetic devices.

## Materials and Methods

### Cloning

LOV constructs were either cloned from genomic DNA (*Methylocystis* Rockwall, kindly provided by Lisa Stein, University of Alberta) or synthetic DNA codon optimized for *E*. *coli* expression (*Methylocystis rosea* and *Methylocystis* Rockwall). Complete DNA sequences of McLOVn, McLOV-TF and McLOVr are provided in supporting information (S1 Sequence). Constructs were cloned by PCR amplification. Following amplification, PCR product was run on a 1% agarose gel to verify PCR product. PCR product was subjected to gel purification prior to digestion with *Xho*1 and *Nco*1 restriction enzymes. Digested DNA was ligated into a 6-His Parallel vector with ampicillin resistance. The resulting ligation reaction was transformed into DH5α *E*. *coli* cells and grown on LB-agar plates containing 200 μg/ml ampicillin. DNA was then isolated via miniprep (Promega). Isolated DNA was confirmed to contain insert by a restriction digest test prior to DNA sequencing (genewiz).

### Expression

Proteins were expressed in *E*. *coli* BL21(DE3) cells grown at 37°C. After the cells reached a density (OD_600_) greater than 0.5, the temperature was reduced to 18°C, and protein expression was induced with 200 μM IPTG. Cell pellets were harvested by centrifugation and stored in buffer (50mM Hepes, 100mM NaCl 10% Glycerol, pH 8) at -80 C.

### Purification

Cell pellets were thawed at room temperature prior to sonication. Cell lysate was clarified via centrifugation at 16,000 rpm to remove cellular debris. Lysate was then applied to a 5 ml Ni-NTA affinity column to isolate the His_6_ tagged protein; the protein was washed and eluted with 20 mM and 300 mM imidazole, respectively. The eluted protein was then incubated with TEV (Tobacco Etch Virus Protease) overnight to remove the His_6_ affinity tag. Cleaved protein was buffer exchanged to eliminate residual imidazole, then subjected to another round of affinity purification to eliminate His_6_-TEV. Finally, the desired protein was isolated with size exclusion chromatography (Superdex 75 GE-Healthcare) prior to concentration to 5–10 mg/ml characterization.

### Photochemical characterization

All experiments were conducted in a quartz cuvette with a pathlength of 1.0 cm. The absorbance was measured at 450nm and 478nm to follow recovery from the light state back to the dark state after photobleaching. Prior to kinetic characterization dark-state samples were illuminated with a 5 mW 473 nm blue laser until a steady-state light/dark ratio was achieved. Recovery was measured at 0.5 s intervals, using the absorption values recorded at 450 and 478 nm. All kinetic traces were corrected for baseline drift by subtraction of the absorbance at 600 nm.

### Eyring and Arrhenius analyses

All protein constructs were characterized with temperature dependent kinetics ranging from 15–36°C followed by Arrhenius and Eyring data analysis to extrapolate rate constants, activation energy, and entropy of activation. All kinetic studies followed procedures outlined above.

### Base catalysis

Base catalysis and determination of solvent accessibility factors were conducted as outlined in Pudasaini et al. [7]. We briefly summarize the methodology here. Constructs were studied for their response to base catalysis by varying the concentration of imidazole within the range of 0–150 mM. We note that higher concentrations were tested, but in many cases led to protein instability. Kinetic parameters were extracted by measuring the absorbance at 450 and 478 nm as outlined above. A solvent accessibility factor (a) was calculated using methods published previously*[34]*. Briefly

rate=a*(k[adduct][base]) (1)

The slope of a plot of k vs. base concentration is equivalent to:

$$\frac{slope}{\left[adduct\right]}=a$$ (2)

The slope was calculated using the concentrations of both protein and imidazole in units of Molarity. All calculations were made with a protein concentration of 10 μM.

### Solvent isotope effect measurements

All SIE experiments were conducted by first exchanging H_2_O buffer (50mM Hepes, 100mM NaCl 10% Glycerol, pH 8) for an equivalent buffer prepared with D_2_O. Buffer exchange was completed via 3 successive 10-fold dilutions into D_2_O buffer followed by concentration to 5–10 mg/ml. The samples were than analyzed for their rate constants via the methodology defined above. SIE are reported as k_H_/k_D_.

## Supporting Information

S1 Sequence Complete DNA and protein sequences for all constructs cloned from *Methylocystis* are provided. (PDF)
