# Supplementary material for: Short LOV Proteins in Methylocystis Reveal Insight into LOV Domain Photocycle Mechanisms
Source: PLoS One. 2015 May 1;10(5):e0124874. doi: 10.1371/journal.pone.0124874 (PMC4416707; doi:10.1371/journal.pone.0124874)
Supplement: S1 Sequence — (PDF) [file pone.0124874.s001.pdf]

DNA and Protein Sequences for McLOVn, McLOVn-TF and McLOVr

>McLOVn genomic DNA
ATGTTTGTCGTTCAAAAAGACAACGGGATCATCCCGGCGATCCTGACCCAGATTCTGGACT
CCTGCGTCAACGGCGCCACGCTCGCGGATCCCGATATGGAGGATGCGCCCATCGTTTAC
GCCAACAAGGCTTTCGAGGACATGACCGGCTACTCGCAGGAGGAGATCATCGGCAGGAAT
TGCCGCTTTCTGCAAGGACAGGACAGCGATCAGGAGGGAATCGGGATCTTGCGCGAAGC
GCTCTCGAAACACGAGAAGGTCGAAGTGACGCTCAGAAATTACAAGAAGAATGGGGAGTT
GTTTTTCAATAAACTTAATATAACGCCACTACTCGATAACAAAGGAAATGTGATATATTATCT
TGGCGTTCAGTATGACGTGACCGAGTTGGTCCGGGCGGAAGAAGAGATCAACCGGCTGG
GTAGCCGCCTGAAGGTGCTGGAAGAGGCT

>McLOVn encoded protein
MFVVQKDNGIIPAILTQILDSCVNGATLADPDMEDAPIVYANKAFEDMTGYSQEEIIGRNCRFLQ
GQDSDQEGIGILREALSKHEKVEVTLRNYKKNGELFFNKLNITPLLDNKGNVIYYLGVQYDVTEL
VRAEEEINRLGSRLKVLEEA

>McLOVn-TF genomic DNA
ATGCACAAGATCGTCGTGGCCGGCCTGGACGGCTGTCTGGGCTCCGCTTTTCTCGGATTG
ACCGATCTTCTGACGCTCGCGCGGCGGGCGATCGGCGGCGCTTTGGCTCCATCCGATCA
GGACTCCGGCGGCGGAGACGCGGACTTCTGCGTCGTCACGGCCAGCGCGACCGGGCGT
CCCGTGCGGGACGGCTCGGGCGCGACTTTCGAGGTCGAGACCTCTTTCGAGGAAGTCAC
GACATGCGACGCCGTTATCGTGCCGAGTTTCGCGCCCGATCCCAACGGCAAGCCGCCGG
ACATGTCGGCCCACGCCGCAGCCGCAGCATGGTTGCGCCGGCATCATTCCCGCGGCGCT
CTTATCGGGGGTTGCGGTTCTGGCGTCTTCCTTCTCGGCGAGGCGGGGTTGCTCGAAGGA
CGTCGTTGCACGACCAGCCGATGGCACCACGAGGAGCTGAAGCAGCGCTATCCGAGAGC
CGACACGGCCTGGGGCGCAAGGTTGATCGACGATCGCCGAGTCGTCACGGCGGCGGGA
CCTCTCTCCTGGATCGACGTGGCGCTGCACGTGATCCGGACGCTCTGTGGACCGGACGC
GGGCCGAATCGCAGCTGACTTCACTCTTGGCGAATCGGCGCCCGCGAAGGGCGGCATAC
GCGGGGCCGCCTACAATGTGAGCGTGCTCGGCGACAGCTCCGATTCGTTTCTTTCCGAGG
CCGAGCGAATCGTCCGGCAATCTGACGCCGCCTTCAACGCTCAGGATCTCGCGCGGGCG
CTCTCTACGTCGGAGCGCACGCTGCACCGAAGGCTGAAACAGGCCTGCGGCGAGTCGCC
CAAGACTTTCATCGACCGCATCCGCGTCGAAACCGCCCGAATGCTGCTGGAAACCAGCGT
CAAGCCCGTGAAGGAGCTGGCGGCCAGCGCCGGATTTATCGACGAGGCGAGCTTCCGCC
GCGCATTCCGCCGCTTCACCGACATGGCGCCGAGCGCCTATCGGGTCTGGGCCAAATCG
AAGAGCCAGGACAAGGCGCAGATGTTTTCCGTTCGCAAGGATTCGGAGATCATCCCCGAG
ATTTTGACGACGATCCTGGATACTTGCGTCAACGGCGTCACCCTGACCGACCCCGATCTA
GAAGATGCGCCAATTGTTTACGCGAACAAGCGATTCGAGGACATCACGGGCTATTCGGTC
GCCGAGATCATCGGCCGCAATTGCCGTTTCCTGCAGGGGCAAGATCGCGACCAAGAGGG
GCTTCGTCGACTGCGCGAGGCCATCAGCAACCGACAGGCAATCGAAGTGACTCTGCGCAA
CTACCGCAAGGATGGCGCGCTGTTTCACAACAAGCTCAACATTACGCCGCTTTTCGACGC
GCAAGGTCAGCTCATCTACTTCCTCGGCGTCCAATATGACGTCACGGACCAGATCCGCGC
GGAAACGGAGATCGGCGACCTCAAAGCCAAGCTTCACTCTCTGGCT

>McLOVn-TF encoded protein (337-TF in blue)
MHKIVVAGLDGCLGSAFLGLTDLLTLARRAIGGALAPSDQDSGGGDADFCVVTASATGRPVRD
GSGATFEVETSFEEVTTCDAVIVPSFAPDPNGKPPDMSAHAAAAAWLRRHHSRGALIGGCGS
GVFLLGEAGLLEGRRCTTSRWHHEELKQRYPRADTAWGARLIDDRRVVTAAGPLSWIDVALH
VIRTLCGPDAGRIAADFTLGESAPAKGGIRGAAYNVSVLGDSSDSFLSEAERIVRQSDAAFNAQ
DLARALSTSERTLHRRLKQACGESPKTFIDRIRVETARMLLETSVKPVKELAASAGFIDEASFRR
AFRRFTDMAPSAYRVWAKSK**SQDKAQMFSVRKDSEIIPEILTTILDTCVNGVTLTDPDLEDAPI**

VYANKRFEDITGYSVAEIIGRNCRFLQGQDRDQEGLRRLREAISNRQAIEVTLRNYRKDGALF
HNKLNITPLFDAQGQLIYFLGVQYDVTDQIRAETEIGDLKAKLHSLA

>McLOVr codon optimized DNA
ATGTTTGTGGTGCAGAAGGACGATGGCATCATCCCGAAGATCCTGACCCAGATCCTGGAC
AGCTGCGTGAACGGCGTGACCCTGGCAGATCCGGATCAGCCGGACGCACCGATCATCTA
CGCCAACAAGGCCTTCGAGAGCATGACCGGCTACGGCCAGGACGACATCATTGGCCGCA
ACTGCCGCTTCCTGCAAGGCGACGATCGCGACCAGGAAGGTTTAGCCCCTCTGCGCGAG
GCCATGCGCAAACACGAGCACATCGAGGTGACCCTGCGCAACTACCGCAAGAATGGCGA
GCTGTTCTTCAACAAGCTGAACATCACCCCGCTGTTAGACAGCCGTGGCGCCGTGATCTA
CTACCTGGGCGTGCAATACGACGTGACCGAGCTGGTTCGCGCCGAGGTGGAGATCAACC
GCCTGGGTCAGCGTCTGAAGGCCCTGGAGAAAGCCTAA

>McLOVr encoded protein Accession number WP_018406994
MFVVQKDDGIIPKILTQILDSCVNGVTLADPDQPDAPIIYANKAFESMTGYGQDDIIGRNCRFLQG
DDRDQEGLAPLREAMRKHEHIEVTLRNYRKNGELFFNKLNITPLLDSRGAVIYYLGVQYDVTEL
VRAEVEINRLGQRLKALEKA
